# Improving the self-assembly of bioresponsive nanocarriers by engineering doped nanocarbons: a computational atomistic insight

**DOI:** 10.1038/s41598-021-00817-2

**Published:** 2021-11-02

**Authors:** Mohammad Khedri, Nima Beheshtizadeh, Reza Maleki, Thomas J. Webster, Sima Rezvantalab

**Affiliations:** 1 Computational Biology and Chemistry Group (CBCG), Universal Scientific Education and Research Network (USERN), Tehran, Iran; 2 Department of Chemical Engineering, Amirkabir University of Technology (Tehran Polytechnic), 424 Hafez Avenue, Tehran, Iran; 3 Department of Tissue Engineering and Applied Cell Sciences, School of Advanced Technologies in Medicine, Tehran University of Medical Sciences, Tehran, Iran; 4 Regenerative Medicine Group (REMED), Universal Scientific Education and Research Network (USERN), Tehran, Iran; 5 Department of Chemical Engineering, Northeastern University, Boston, MA USA; 6 Renewable Energies Department, Faculty of Chemical Engineering, Urmia University of Technology, Urmia, 57166-419 Iran

**Keywords:** Biomedical engineering, Two-dimensional materials, Computational science, Computational nanotechnology, Drug development

## Abstract

Here, molecular dynamics (MD) simulations were employed to explore the self-assembly of polymers and docetaxel (DTX) as an anticancer drug in the presence of nitrogen, phosphorous, and boron-nitrogen incorporated graphene and fullerene. The electrostatic potential and the Gibbs free energy of the self-assembled materials were used to optimize the atomic doping percentage of the N- and P-doped formulations at 10% and 50%, respectively. Poly lactic-glycolic acid (PLGA)- polyethylene glycol (PEG)-based polymeric nanoparticles were assembled in the presence of nanocarbons in the common (corresponding to the bulk environment) and interface of organic/aqueous solutions (corresponding to the microfluidic environment). Assessment of the modeling results (e.g., size, hydrophobicity, and energy) indicated that among the nanocarbons, the N-doped graphene nanosheet in the interface method created more stable polymeric nanoparticles (PNPs). Energy analysis demonstrated that doping with nanocarbons increased the electrostatic interaction energy in the self-assembly process. On the other hand, the fullerene-based nanocarbons promoted van der Waals intramolecular interactions in the PNPs. Next, the selected N-doped graphene nanosheet was utilized to prepare nanoparticles and explore the physicochemical properties of the nanosheets in the permeation of the resultant nanoparticles through cell-based lipid bilayer membranes. In agreement with the previous results, the N-graphene assisted PNP in the interface method and was translocated into and through the cell membrane with more stable interactions. In summary, the present MD simulation results demonstrated the success of 2D graphene dopants in the nucleation and growth of PLGA-based nanoparticles for improving anticancer drug delivery to cells, establishing new promising materials and a way to assess their performance that should be further studied.

## Introduction

Nanocarbons of various dimensions (graphene, fullerene, nanodots, etc.) provide for a broad number of candidates in medicine and biology^1^ as well as other technologies (e.g. in electronic devices^2^, batteries^3^, sensors^4^, etc.) due to their appealing properties such as low cost, low toxicity, and surface fuctionalization. Graphene, as a lattice of a carbon allotrope, due to its physicochemical properties (e.g. thermal and electrical conductivity, flexibility, and transparency) has also recently attracted wide attention in biomaterials^5^ and medicine^6,7^. Its growing applications in the biomedical field include the synthesis of organic and inorganic nanoparticles, scaffolds^8,9^, nanocarriers^10^, nanofibers^11,12^, and hydrogels^13,14^. Fullerene, another symmetric carbonaceous nanomaterial that is a closed zero-dimensional unit with a cage shape^15^, has attracted further attention with broad applications in biomedical applications^16–18^, as batteries^19,20^, etc. Fullerenes unique properties such as highly tenability, large surface-to-volume ratios and high electron affinity provides a broad range of applications^21,22^. The size of the cage depends on the number of carbon atoms (e.g. C60, C76, and so on) with interesting properties such as a rigid structure in solvents^23^. Greater control on the properties of graphene, fullerene, and other carbonaceous nanomaterials has been achieved by substituting carbon atoms with other elements as nitrogen, boron, silicon, etc.^24–28^ Graphene, fullerenes, and their dopants as well as other derivatives have already been used for improved anticancer drug delivery^29–34^. These nanocarbons have excellent characteristics such as high specific area, high mechanical properties and low charge scattering that make them suitable for cancer therapy as either a drug/gene carrier and photothermal agent or as a biosensor^35^.

From another point of view in carbon-based synthesis science, researchers have reported the use of graphene as a seeding agent for the self-assembly of materials and also as a size controller that can produce ultrasmall nanoparticles useful for penetrating tumors, cells, etc. For instance, Deshpande et al.^36^ reported that the use of graphene induces one-dimensional ordering of 10,12-pentacosadiynoic acid in a ultrahigh vacuum. In other research, their team used graphene as a template for the self-assembly of a sub-10 nm layer of 10,12-pentacosadiynoic acid that can assemble into one-dimensional arrays^37^. They claimed that graphene can serve as an intermediate for parallel nanopatterning^38^. Further, Vo et al.^39^ reported the use of nitrogen (N)-doped graphene in the synthesis of one- (1D), two-(2D), and three-(3D) dimensional metamaterials. Their results revealed that using N-doped graphene nitrogen-substitution prompts hydrogen bonding interactions. While hydrogen bonding promotes 1D and the planner growth of nanocarbons, van der Waals (vdW) and π-stacking interactions from the surfaces create micro-scaled crystals. It shows that the physical and optoelectronic properties of these microcrystals can be controlled from nanoscale components.

In biomedical applications, polymeric nanoparticles (PNPs) can be utilized to deliver a wide range of drugs and for improved diagnostics. Indeed successful drug delivery is the ultimate goal of PNPs for which various techniques and strategies have been examined to boost delivery efficiency and efficacy. For example, the stability of the particles is an important issue that has led to the establishment of self-assembled amphiphilic copolymers^40^. Furthermore, decorating the surface of PNPs with stealth sheath polymers and/or targeting ligands and moieties can avoid off-target delivery^41^. These strategies and techniques need further consideration to produce PNPs with more predictable and defined properties^42^. Despite the promising developments in this field, control of polymeric NP size is considered a major remaining challenge.

Most reports on the use of ultra-small NPs (< 30 nm) in biomedical research include inorganic and metallic particles (e.g. iron oxide, gold, etc.)^43^. Researchers have published a few reports on the methods and strategies to achieve this range, e.g. in situ polymerization^42,44^, but these methods have some disadvantages like a large number of residual monomers and they need surfactants^45^. Researchers have attempted other new strategies to control nanoprecipitation events by control of NP charge^46^ or its modified versions^47^. However, manipulation of surface charge can change the NP behavior and performance in the biological environment. Since it shifts their time in blood circulation due to interactions with proteins (the so called “protein corona” effect) and/or their recognition by macrophages for clearance from the body.

Moreover, surface charge is a decisive feature in cellular uptake and also stimulation of the immune system due to their interactions with cell membranes. Another attempt to control nanoprecipitation is to control the interactions of polymer chains by the control of momentum and subsequently NP formation. This process was achieved by carrying out NP precipitation in very small (micro-scale) channels as microfluidic systems^48^. In this method, the mixing for nanoprecipitation is accomplished only by molecular diffusion^49^. Microfluidic systems in the synthesis process can be classified into two general types based on the type of reagent flow: segmented or droplet-based flow and continuous-phase (jet) flow microfluidic systems^50^. Continuous-phase flow microfluidics has been utilized in the production of nanocarriers while the other type generates micro-sized particles. It is of great interest to understand the size control phenomena that happens at the interface of flowing streams leading to nanoparticles.

Nowadays, simulation methods are valuable tools for the evaluation and even prediction of the properties and performance of materials and structures with various components as well as their interactions with other compounds and environments. Moreover, simulations predict logical explanations for experimentally inaccessible structures and phenomena. Furthermore, the mathematical model before experimental evaluations are cost-effective and cuts the costs of unnecessary analysis. Additionally, it can decrease the health risks of working with hazardous agents like anticancer drugs^51^. In this regard, recently, we used molecular dynamic (MD) simulations and declared how interfacial effects control the solvent diffusion and displacement that subsequently controls the formation of particles and their sizes^52^.

Here, by using atomic-scale simulations, we investigated the impact of various nanocarbons present in the assembly of amphiphilic copolymers and consequently the formation of polymeric nanoparticles (PNPs) at the interface of organic-nonorganic fluids. In the current work, riboflavin (RF), as a promising active targeting ligand^53,54^, was used in the simulations where RF-terminated PLGA-PEG-based PNPs were employed in the self-assembly. Previously, the density and orientation of the PLGA-PEG-RF strands in PNPs were optimized using MD simulation^55^. We utilized random and interface approaches to mimic the bulk and microfluidic circumstances, respectively. Moreover, diverse nanocarbons were employed to predict their effect in the self-assembly process and evaluate the properties of the final particles. The present results offer deep insights into the employment of nanocarbons in microfluidic-based synthesis for improved anticancer drug delivery.

## Results and discussion

### Self-assembly of drug-loaded PNP

#### Doping optimization

The modification and doping of nanocarbons is an effective method to improve their chemical and physical properties. However, it should be noted that further replacement of carbon atoms in the structures can reduce their overall morphology, performance, and properties. Therefore, it is of great importance to find an optimized combination of carbon and doped atoms prior to further investigations. Charge transfer due to doping in the structure happens in nanomaterials^56,57^. For instance, using density functional theory (DFT) method, Chi et al.^58^ found that the net electron transfer from the Al-doped graphene to H_2_CO is four times more than that in the intrinsic graphene. They concluded that Al-doped grapheme can be used as a chemical sensor for detection of H_2_CO. In another study, Thakur et al.^59^ presented that N-doped fullerenes have the highest level of polarizability and hyperpolarizability to the presence of electronegative nitrogen atoms surrounded by positive carbons. However, the phosphorous neighboring carbons have partially negative charges that can be caused by the differences in their electronegativity. Thakur et al.^59^ studied the effect of substituting atoms (e.g., nitrogen and phosphorus) in the properties of fullerenes and reported that the highest polarizability of fullerene-dopants are related to non-metal doped samples for N-doped components. With N-doping into the cage structure of fullerene, charge transfer occurs between the N and C atoms, resulting in unbalanced charge distribution in the azafullerene. This makes the N − C complex site in N-doped fullerene preferential for electrophilic and nucleophilic attack.

**Figure 1:**
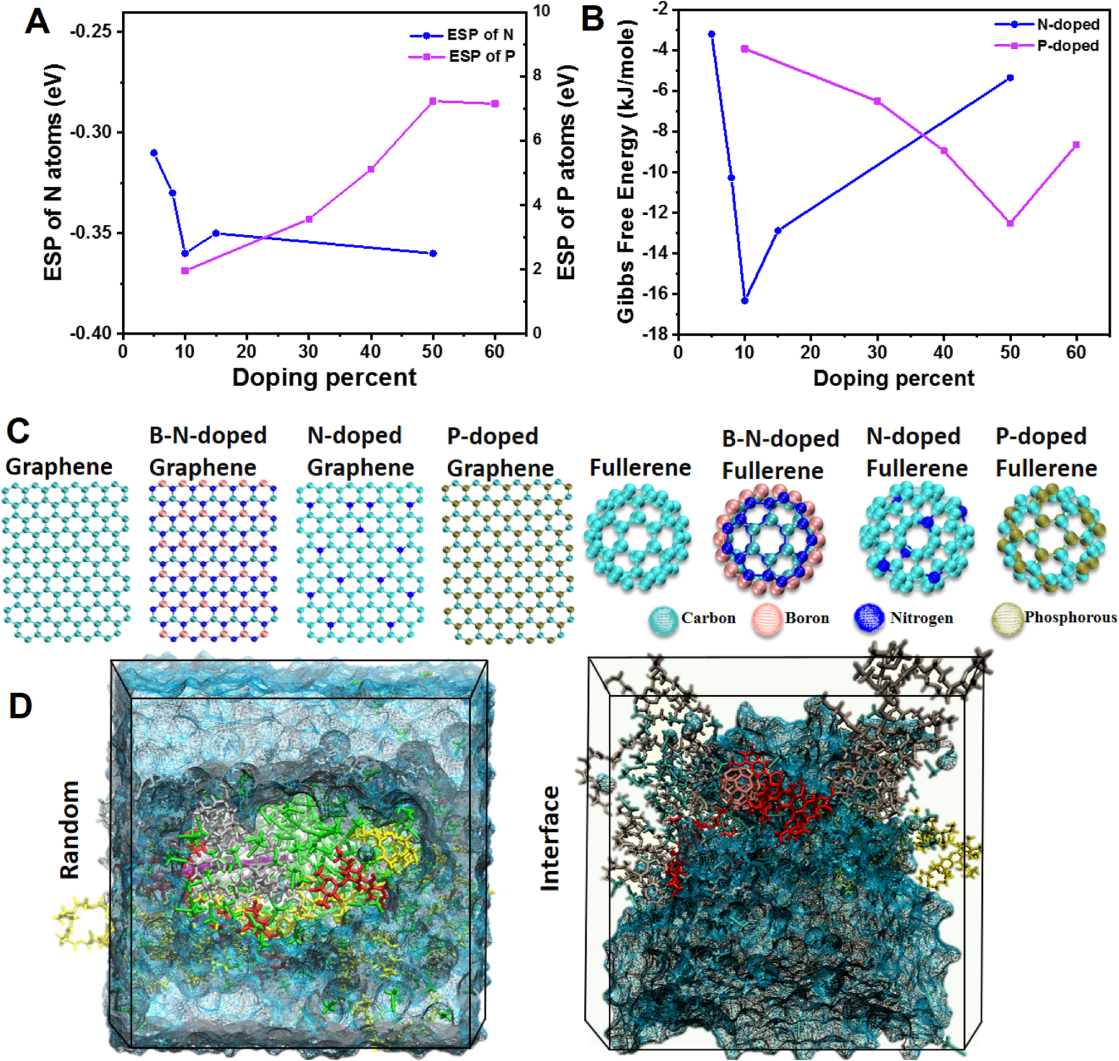
**(A)** Charge transfer of N- and P-doped graphene. (**B)** Gibbs free energy results for the self-assembly process of polymers in the presence of doped graphene with varying amounts of doped molecules. Consistent with part A, the highest charge transfer in the structure leads to the formation of the most stable polymeric NPs. (**C)** The molecular structure of carbon nanomaterials used in the seeding of polymeric self assemblies. (**D)** Representation of the simulation methods; random and interface. Blue, green, and red molecules represent water, ACN, and DTX, while RF-conjugated polymer chains are yellow and PLGA-PEG strands are grey. The graphene nanosheet is purple (random method) and fullerene is pink (in the interface method).

In the current study, various doping atoms have been considered. As we know, nitrogen has the highest electronegativity (3) followed by carbon (2.5), phosphorous (2.1), and finally boron (2.0). The more the doping percentage used, the more the unbalanced charge distribution on the surface. Figure 1.A shows the effect of doping in the polarizability of the nanosheet surface charge. By increasing the atomic N-doping percentage above 10%, the electrostatic potential (ESP) of nitrogen atoms decrease and meet the maximum at 50% doping. In other words, nitrogen atoms attract more electrons to the surface and become negative.

In the case of the P-doped nanosheets, an increase in the doping percentage, leads to an increase in polarizability of the structure, i.e. phosphorous atoms take more positive charges. These observations are due to the lower electronegativity of the P atoms in comparison with carbon atoms. Therefore, carbon atoms attract more electrons, and phosphorous atoms on the surface become positive. All the observations are can be attributed to the differences in element electronegativity. N-dopants exhibit a different behavior where nitrogen atoms behave like acceptors while introducing P atoms change the whole structure's electron distribution and P atoms behave as donors and carbon atoms as acceptors. It has been shown that the N–N bond in the structure of N-doped graphene reduces the stability of the architecture^60^. According to these results, the N-doped graphene at 10% and 50% have almost similar ESP values as nitrogen atoms, thus, to avoid instability of the nanocarbon, we consider 10% doping as the optimal case for self-assembly.

Next, to assure these optimizations, the Gibbs free energy for the assembly of 8 PLGA-PEG and 2 PLGA-PEG-RF strands was calculated in the presence of various N-and P-doped graphene nanosheets (Fig. 1B). The polymers employed in the self-assemblies were set at PLGA-PEG: PLGA-PEG-RF at a 8:2 ratio which was detected as the most stable composition in previous studies^52^. In agreement with charge analysis, the lowest level of Gibbs free energy was obtained in the presence of N-doped (10%) and P-doped (50%) formulations. The lower the Gibbs free energy of the self-assembly, the more stable and spontaneous the process. However, the only option for the BCN structure is the 33% N-doped with ESP values as N: −1.7, B: 1.15, C: 0.55.

The same compositions for the fullerene-based materials were selected and the ESP analysis is presented in Table 1. Interestingly, 2D doped nano-objects have lower charge transfer in the fullerene family with the same composition. For example, nitrogen atoms have an average ESP: −0.36 in the N-doped graphene, while the N-atoms have an ESP of −0.15 for the N-doped (10%) fullerene. This can be attributed to the steric hindrance in flat objects wherein the case of fullerene-based materials atoms have more available space, and subsequently, less charge transfer occurs on the surface of structures.

**Table 1:** The effect of doping on the electrostatic potential (ESP) distribution of fullerenes.

N-doped			P-doped			BCN		
Percent	Element	ESP	Percent	Element	ESP	Percent	Element	ESP
10	Carbon	0.02	50	Carbon	−0.6	50	Carbon	0.16
	Nitrogen	−0.15		Phosphorous	0.6		Boron	0.98
							Nitrogen	−1.14

Figure 1C represents the topology and the structure of the nanocarbon used in the assembly of the polymers. As shown in each category (sheet or spherical nanomaterial), simulations were performed with the carbon-based nanomaterial and their derivatives as dopants including the atomic replacement of carbons (Table S1). The two simulation styles corresponding to nanoprecipitation with bulk and microfluidic circumstances are hereinafter defined as random and interface methods (Fig. 1D), respectively. In the random style of the polymer strands, drugs, carbon nano-objects, and ACN molecules were added randomly and uniformly in between the water molecules in the simulation boxes. However, in the interface mode, polymer chains together with DTX and nanocarbon were brought into contact with water molecules at the interface. The assembly of polymers was investigated under different simulation methods with the presence of each nanocarbon.

#### Effect of doping

**Figure 2:**
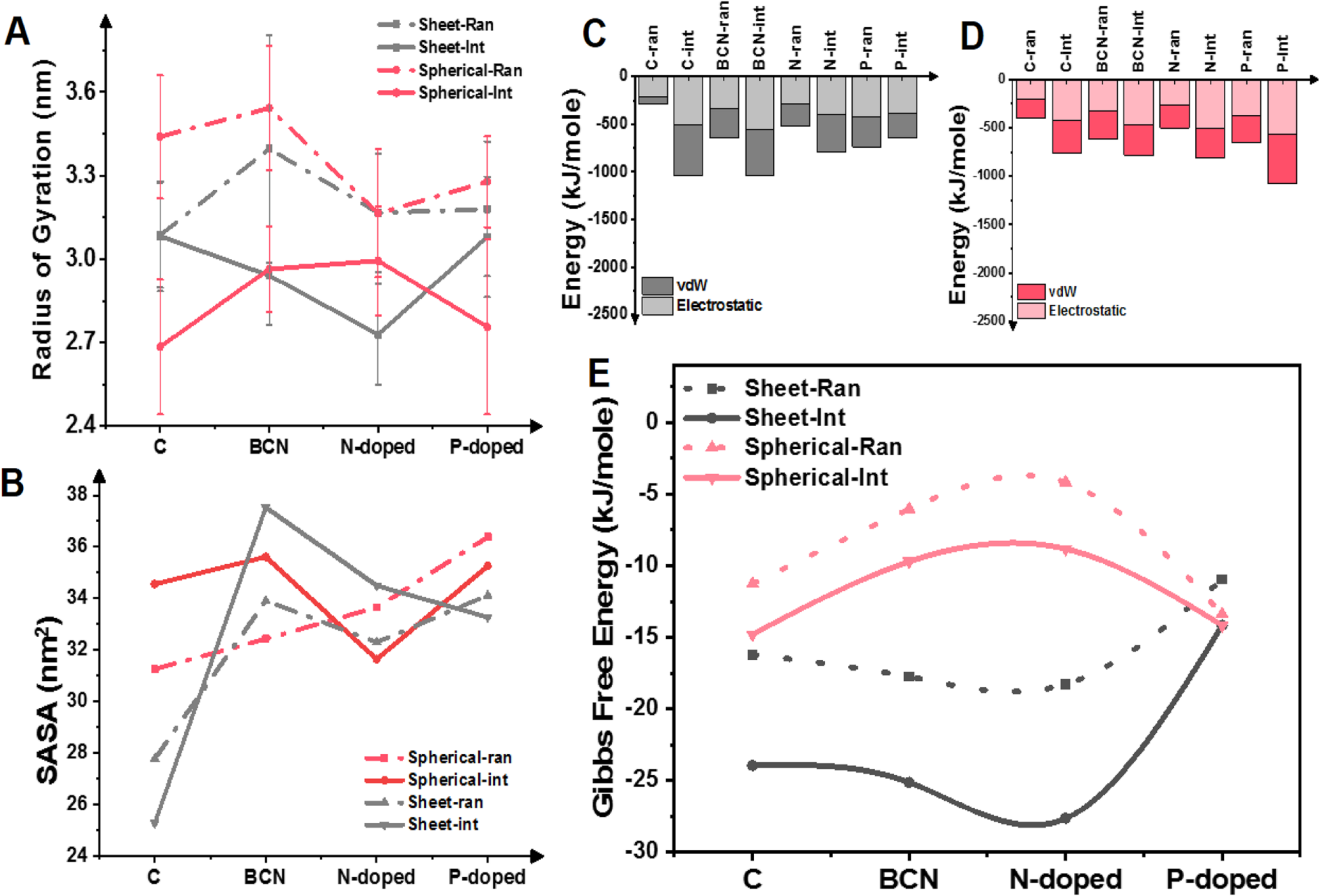
Initial analysis of PNPs in the presence of carbon-based nano-objects. **(A)** Results of mean Rg calculated in each simulation show that the presence of nano-objects in the interface method gives rise to smaller NPs in comparison with their peers in the random style. (**B)** The solvent available surface area of the assemblies formed in the presence of nanocarbon. (**C,D)** Interaction energies of polymeric strands within the micelles are plotted for nano-sheets and spherical fullerene-based materials, respectively. As is shown, the vdW interaction is the dominant interaction in the assembly process. (**E)** Gibbs free energy as the absolute stability criteria have been calculated for samples. In agreement with Rg results, NPs that are assembled in the interface of ACN and water are more stable and have lower energy levels. Moreover, NPs produced in the presence of graphene and its dopants are more stable than NPs assembled in the presence of fullerene and its dopants.

The initial analyses were performed to monitor the properties of the resultant PNPs under different conditions. To this end, the radius of gyration (Rg) of each PNP as a function of time was calculated and represented as the average amount over the simulation time (Fig. 2A). Recalling that Rg is the radius of accumulation resulting from the accumulation of molecules, Rg results show the energy intensity between the molecules and the stability of the system^52^. It should be mentioned that hereinafter red and grey diagrams illustrate the presence of graphene- and fullerene-based nanomaterials in the simulation whereas solid- and dashed-lines outline the interface and random method. According to the plots, using graphene-dopants, especially nitrogen-doped samples, promote the formation of more tight PNP while using fullerene-dopants in the interface method, disturbs the assembly and produces PNP with more scattered branches.

The solvent available surface area^10^ of the PNPs is affected by the doping (Fig. 2B). Due to the presence of carbon atoms, pristine nanocarbon provided the lowest surface area toward water molecules and consequently attracted the hydrophobic segments of copolymers. Doping of the architectures with other atoms changes their hydrophobicity. Consistent with Afshar et al.^61^, our results confirm that N- or P-doped graphene and fullerene have higher hydrophilicity in comparison with pristine graphene and fullerene. The highest SASA values are related to the BCN structures that have the lowest amount of carbon atoms.

#### Effect of the simulation method

In each category (graphene- or fullerene-based calculations), assembled PNPs at the interface of the organic/inorganic solvents had a smaller Rg size compared with bulk samples. This is indicative of diffusion-controlled mass transfer through the interface layer that leads to the smaller diameter of PNPs when compared with their peers in the random method. This observation is consistent with previous findings that the assembly of polymer chains at the interface of two fluids gives rise to smaller and denser particles. The root-mean-square-deviation (RMSD) assessment verifies the observed trends for all cases (Figure S1) where the fluctuation of each particle formation in the interface method is lower than its peer in the bulk method. Another striking observation is that the assembly of polymers with graphene in the random and interface method produces PNPs of almost the same size. This can be contributed to the planner geometry of the nanocarbon that makes its transfer easier through phases and seeds the assembly of polymers in the same conditions.

To further characterize the PNPs, the energy level of the PNPs was pursued. The total potential energy is a function of bonding and non-bonding interactions. Three main interactions are dominant in the formation of NPs: vdW and electrostatic energies and hydrogen bond interactions, where, the total energy is vdW and electrostatic resultant energies. So, for this system, the main parts of energy are vdW and electrostatic energies, which are important criteria for stability. Due to the fact that the difference in the number of hydrogen bonds (because all cases had the same polymeric compositions) contributed to low energy interactions, its impact on stability has been ignored in comparison with vdW and electrostatic energies. Here, we calculated the average interaction energies over the assembly time, and Fig. 2C,D represents the results for PNP in the presence of the graphene and fullerene family, respectively. As is clear, PNP prepared in the interface style have consistently larger interaction energies than those assembled in the random style, except for P-doped graphene. We can attribute the issue to the chemical composition of nano-objects that will be discussed in the following sections. Moreover, the results clearly showed that the self-assembly process is driven by electrostatic interactions, especially since its contribution is higher than vdW interactions for the interface samples. It is worth mentioning that in the line with charge transfer and polarizability of the nanocarbon, due to the doping, electrostatic interactions grow. For instance, with 10% N-doping in the graphene nanosheet, the numerical value of the electrostatic energy for the assembly increased from ca.217.17 kJ/mole to ca. 291.29 kJ/mole. Similar and even larger increases are obvious with BCN and P-dopants that can be attributed to the higher percentages of doping. Similar trends are noticeable for fullerene-based samples.

To substantiate the observations even further, Gibbs free energy of the self-assemblies was computed and is presented in Fig. 2E. The Gibbs free energy serves as the absolute criteria of the stability of the PNPs. As shown in the plots, results are in good agreement with previous findings that for all samples, particles prepared at the interface of organic/water solutions have a lower energy level and consequently better stability. The above-mentioned assessments revealed that PNPs simulated in the interface mode are more stable than those modeled in the random method.

#### Effect of geometry

During this study, it has been highlighted that changing the geometry of nanocarbon led to distinctive results in the assembly of polymeric NPs. It is readily apparent from Fig. 1 that utilizing graphene derivatives in the interface method led to PNPs with relatively smaller Rg sizes and lower Gibbs free energy levels. This can be attributed to their two-dimensional (2D) and planar structure that provides for higher surface area. In this regard, we have calculated the surface area to volume ratios for all the carbon-based nanoobjects to quantitatively analyze the results (Table 2). As it is clear, 2D nanocarbon has a higher surface area to the volume ratio compared with their peers of the spherical geometries (0D). It is obvious that the inner surface area of the fullerene cage is inaccessible for polymer strands, therefore, the interactions between polymer strands and nanocarbons that lead to the self-assembly of PNPs is limited. In the presence of graphene-based nanocarbon, the polymer can attack the nanocarbon from both sides. It worth mentioning that doping in the fullerene-based nanocarbon interrupted the stability of the aggregated PNPs around them. For example, N-doped fullerene induced the PNP to have the lowest stability (Fig. 1D) among its peers (both random and interface method). According to the energy diagrams, nevertheless, of N-graphene or N-fullerene, the difference between the interaction energies of the assemblies is negligible. Therefore, the difference in the Gibbs free energy and consequently stability level of the PNPs in the presence of N-graphene or N-fullerene can have an entropic origin, where it is attributed to the geometrical and physical features of the nanocarbon.

**Table 2:** Properties of nanocarbon and the average H-bonds between the drug and micelles for each case.

Geometry	Structure	A (nm^2^)/V (nm^3^) ratio	MSD (nm)		Average micelle-drug H-bonds	
			interface	RANDOM	Interface	Random
Sheet	Graphene	13.33	8.88 ± 4.39	9.24 ± 3.59	5.02 ± 2.20	2.95 ± 1.9
	BCN	13.33	5.25 ± 3.43	6.80 ± 3.96	4.02 ± 2.07	3.03 ± 1.77
	N-doped	13.33	7.22 ± 3.37	6.99 ± 4.05	3.4 ± 1.88	2.91 ± 1.7
	P-doped	13.33	4.53 ± 2.1	6.12 ± 3.47	2.64 ± 1.85	2.5 ± 159
Spherical	Fullerene	4.22	6.93 ± 3.91	5.41 ± 2.84	3.06 ± 1.76	2.26 ± 1.50
	BCN	4.22	4.22 ± 1.96	8.21 ± 4.83	2.85 ± 1.6	3.22 ± 1.87
	N-doped	4.22	4.09 ± 1.64	5.00 ± 2.02	2.29 ± 156	2.73 ± 1.64
	P-doped	4.22	4.53 ± 2.1	4.90 ± 1.75	2.4 ± 2.38	2.99 ± 1.8

Likewise, the planar nanocarbons can easily travel from one phase to another phase compared with spherical compounds. Hence, 2D objects can better induce and seed the assembly of PNP, due to easy movement and hydrophobic attractions in contact with hydrophobic segments of polymers and drug molecules. To quantitatively analyze the diffusion of nano-objects in the simulation boxes, mean square displacements (MSD) were calculated for individual cases. MSD is a distance that an object moves in the course of simulation^62^. In this regard, the MSD values represented in Table 2 confirm the aforementioned facts that 2D nanocarbons can get through longer distances. However, as mentioned before, the interface method limits the diffusion distance to control the assembly. Therefore, there is a subtle trade-off governance between the geometry of nano-objects and the simulation method. In the presently considered cases, the geometry of the nano-object is more dominant and it can be easily understood that planar graphene-based objects have higher MSD values.

As the geometry affects multiple parameters, it also changes the interaction energies of the structures in the assembly process. As mentioned before, electrostatic energy increases by doping the structures for the graphene-based nanocarbon. The increase is also obvious for fullerene-based nanocarbon while the difference for spherical structures, the increase is smaller. As for fullerene in the random style, the electrostatic energy was 201.61 kJ/mole and with 10% N-doping, it surges up to 269.71 kJ/mole. In comparison with graphene (mentioned earlier), the difference between electrostatic energy in the assembly with pristine and dopant fullerene is smaller which can be attributed to the geometrical limitations discussed above. On the other side, vdW energies are higher for the 3D samples in comparison with 2D nanosheets. All of these observations can be attributed to the geometrical differences in flat and spherical architectures.

Another factor in the stability of the NPs is the hydrogen bonds (H-bonds) formed between polymer strands and DTX molecules. Besides the composition of nano-carbons, geometrical availability and the method of synthesis can affect the number of H-bonds. Table 2 outlines the average number of H-bonds in the course of simulations in each case. For instance, for the presence of nanocarbons in the interface method, the highest H-bond numbers correspond to the pristine graphene/fullerene since the H-bonds can form between polymer strands and DTX molecules. However, doping N-atoms in the structure provides H-bonds to form between the nanosheets-DTX and the nanosheets-polymer chains. As a result, the number of H-bonds between the DTX-polymers drops. Furthermore, in the interface method, the H-bonds formed (between DTX-polymers) are higher than the random method, which is additional evidence for the stability of the NPs produced in the interface method (corresponding to the microfluidic method in the wet-lab).

**Figure 3:**
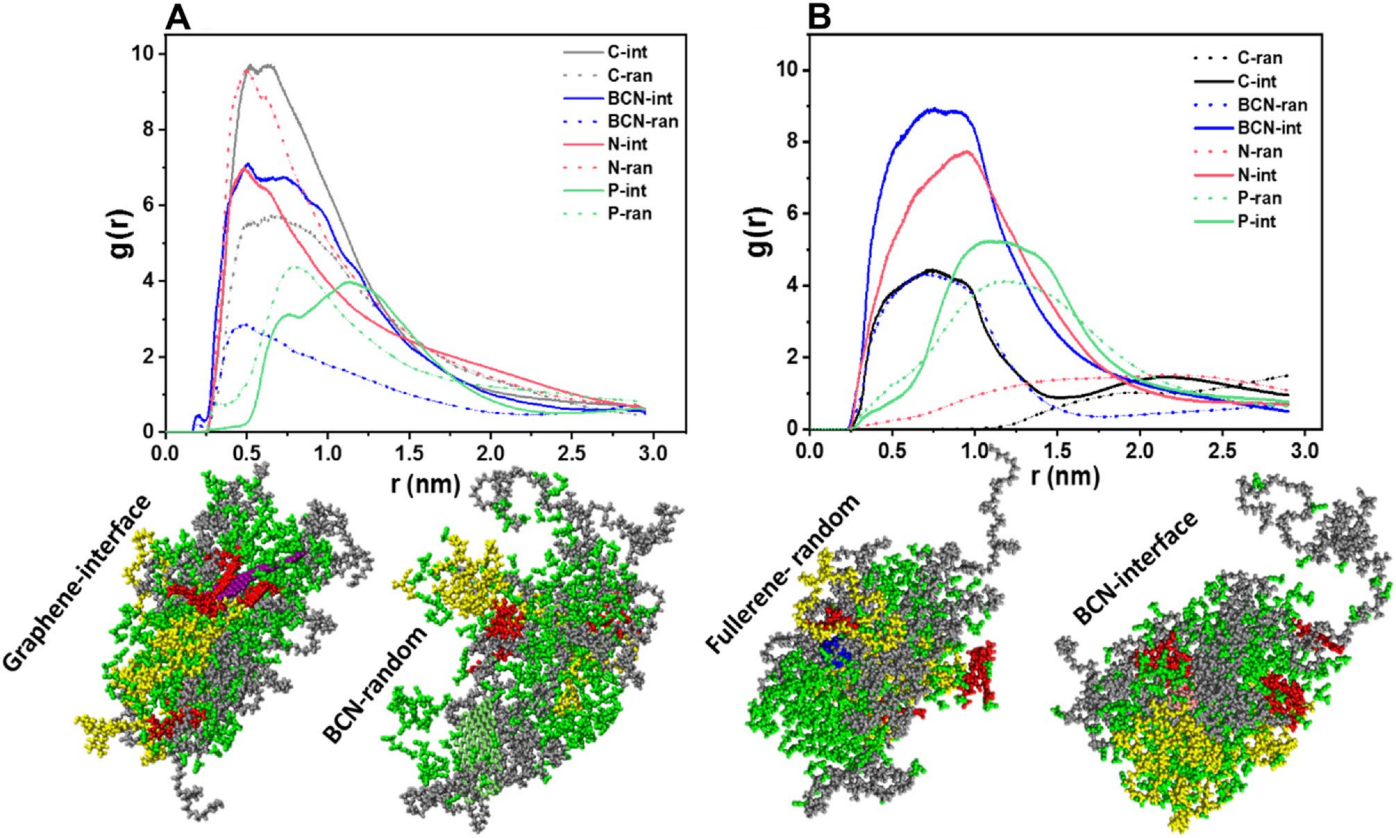
**(A,B)** Radial distribution function (RDF) of the center of mass of PNP in the individual simulations for graphene- and fullerene-based nanocarbon, respectively. In each subgroup, snapshots of the case with the highest and lowest values are provided.

The radial distribution function of DTXs was analyzed and sketched in Fig. 3 together with snapshots of PNPs with the highest and lowest level of RDF in each category. Graphene-induced PNP in the interface method had the highest level of RDF which implies that the DTX molecules are mostly trapped in the center of mass of the NPs (as shown in the snapshot). In contrast, fullerene in the random method aggregates polymers that have the lowest content of DTXs in its center of mass. Noticeably, nanocarbons are in the center of mass and polymer strands are attracted around their structure. These observations clearly show the capability of the present objects to induce self-assembly.

### Lipid bilayer penetration

**Figure 4:**
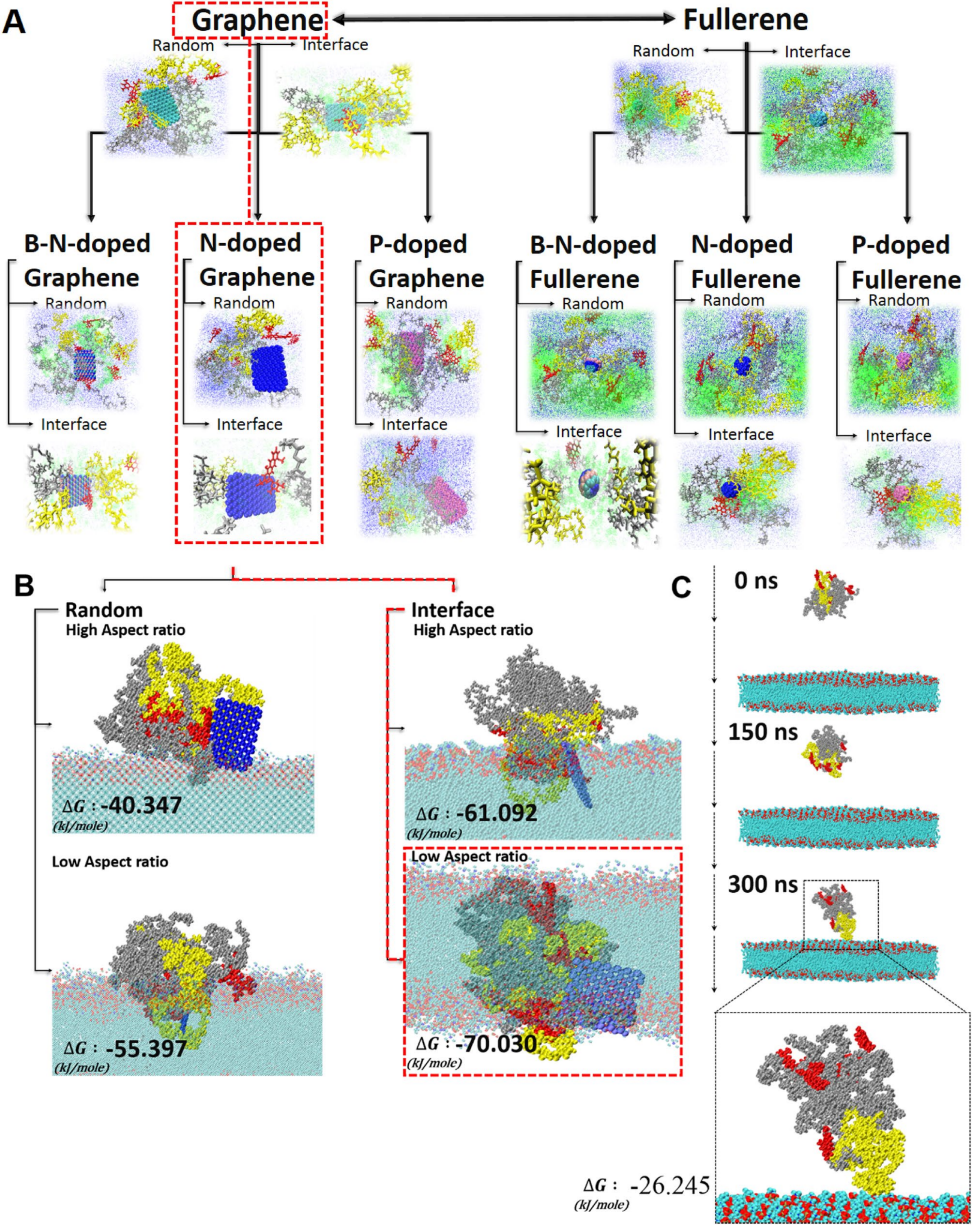
The roadmap for the selection of suitable nanocarbons for the self-assembly of PNPs. **(A)** Assessments done in the first part of the current paper can be presented in a tree diagram where the N-doped graphene provided the best qualities of NPs among its peers. (**B)** Translocation of PNPs produced with N-doped graphene with varying aspect ratios in both methods of synthesis at the end of the 300 ns simulations. (**C)** Stepwise translocation of PNPs prepared in the random method without any nanocarbons.

In the first part of the current study, we considered the effect of the synthesis method and composition on the properties and stability of PNPs. Concludingly, results showed that N-doped 2D graphene (Fig. 4A) assisted in the formation of more stable PNPs with tight architectures. The tree diagram showcases the overall simulations performed in the first part of this study and shows the impact of the nanocarbons on the self-assembly of PNPs.

For drug delivery purposes, PNPs not only should have better properties resulting from their synthesis, but also the prepared PNPs need to penetrate through cancer cell membranes for drug delivery. In this regard, we also explored the effect of the synthesis method and geometrical parameters of the N-doped graphene in the penetration of NPs through a cell lipid bilayer membrane. The N-doped graphenes were designed with the same composition and different aspect ratios to monitor whether the resultant NPs were affected by the physicochemical properties of the seeding agent. In the first part of the paper, the aspect ratio of the nanosheets was 1.2 (termed as a high aspect ratio). Here, in the second part, the aspect ratio is considered to be effective for the performance and seeding of the assemblies. In this context, new PNPs were also prepared with N-doped graphene nanosheets with an aspect ratio of one (termed as a low aspect ratio). The PNPs were induced and prepared in both methods (random and interface). Afterward, the PNPs were transferred to other simulation boxes containing lipid bilayers to monitor their interactions. The Gibbs free energy of the penetration was calculated for the PNPs aggregated around the 10% N-doped graphene (Fig. 4B). The penetration processes were carried out for 300 ns for each individual case. Strikingly, regardless of the synthesis method, PNPs prepared around the N-doped graphene with a lower aspect ratio had a higher affinity toward the cell membrane since the lower the Gibbs free energy, the more spontaneous the interaction (Table S2). Moreover, PNPs prepared in the interface mode had better interactions with the cell lipid bilayer.

The insertion of PNPs was in the direction that the N-doped graphene was perpendicular to the surface of the membrane. Except for the sample seeded with the highest aspect ratio N-doped graphene in the random approach, other samples contacted the cell membrane by the RF-conjugated polymer. The mentioned sample had the lightest level of interaction with the cell membrane that showed the least affinity toward the membrane. We recently showed that RF-ligands were located at the surface of PNPs. Herein, it can be seen that the targeting ligands were oriented toward the lipid bilayer and assisted in targeting the tumor cell. Due to the fact that the RF ligand is a hydrophobic structure, hydrophobic interactions between the RF and cell lipid membrane stimulates the insertion of PNPs into the cell. Nanosheets with a lower aspect ratio promoted more symmetric PNPs that exhibited better insertion into the cell lipid layer. Furthermore, PNPs prepared with the interface approach were tight and had smaller Rg sizes that improved better penetration due to a smaller size as well as a more stable architecture.

Stepwise translocation of the PNP prepared in the random method without any nanosheets as the control group is presented in Fig. 4C. The first interaction of the membrane and PNP is from the RF-conjugated side. However, the Gibbs free energy calculation revealed a higher level of energy (−26.245 kJ/mole which indicates an unstable interaction in comparison with nanocarbon accompanied PNPs). The snapshots show that even after 300 ns, PNP cannot translocate the membrane whereas its peer prepared together with N-doped graphene permeated the cell membrane.

## Conclusion

In the current work, MD simulation was employed to investigate the impact of graphene and fullerene-based nano-objects in the self-assembly of PLGA-PEG polymers for anticancer drug delivery applications. The study was composed of two parts and aimed to mechanistically distinguish the difference between flat and spherical nanocarbon in seeding PNPs. Through the assessment of EPS and Gibbs’ free energy of the self-assemblies with varying percentages of N- and P-dopants, it was identified that the substitution of carbon atoms with 10% and 50% of nitrogen and phosphorous atoms, respectively, gave rise to PNPs with the lowest energy content as stable samples. Due to the higher electronegativity of N- atoms, carbon atoms acted as donors. On the other hand, carbon atoms acted as acceptors using P-atoms.

Next, the aggregation of polymer strands with DTXs was performed using pristine nanocarbons, B-N-, N, and P-doped structures. The energy values showed that N- doped graphene was energetically more favorable for the seeding of PNPs atoms and a small size was obtainable. The method of synthesis (interface or random) creates PNPs with varying properties. Regardless of doping and geometry, the interface method controls the diffusion and prepares PNPs with smaller and more stable structures. The geometry of the nanocarbon was a determinant property since the available surface area to volume ratio of the objects is decisive in attracting and seeding of PNPs. In this context, planner nanocarbon provides a higher surface area to start the self-assembly of polymers. Moreover, the geometry limits the diffusion of the 0D nanocarbon between organic and non-organic phases and consequently their performance.

Indeed, the permeation of PNPs into a cancer cell membrane is the ultimate goal of drug delivery anticancer systems. Therefore, at the final stage, the interaction of the selected PNP (prepared with 10% N-doped graphene) with cancer cells was assessed. The results from the Gibbs free energy translocations led us to choose PNP prepared with the 10% N-doped graphene at an aspect ratio of 1 using the interface method as the best formulation to recommend for further development and improved anticancer applications.

## Molecular dynamics (MD) simulations

### Molecule designs

The current study is considered as a follow-up to our previous paper^52^. Therefore, the optimized ratio of PLGA-PEG:PLGA-PEG-RF (PP: PPR) at 8:2 was considered for the simulations that corresponds to the PNP with 20% wt of the ligand-conjugated polymer. The molecular structure of the polymers was designed using Gauss view 6 software where repeating units of the PLGA copolymer consisted of an equal number of the lactic and glycolic units (L:G ratio 50:50). The graphene and fullerene structures were constructed using Nanotube Modeler software and their derivatives (boron-, nitrogen-, and phosphorous -doped structures) were developed using Gauss view 6 software. In the next step, the Gaussian 09 software (Method: b3lyp and basis set of 6–31 +  + G) was employed to optimize the structure of the carbon-based nanomaterials, acetone (ACN), and docetaxel (DTX). Moreover, the Gaussian 09 software with the ONIOM method was employed to optimize the structure of the polymer chains in three layers: high (b3lyp and basis set of 6-31G +  + G (d,p)), medium (b3lyp and basis set of sto-3 g), and low (PM6).

The charge computational simulations were performed base on density functional theory (DFT) using Gaussian 09 package. We have optimized geometries of pristine and doped graphene at wB97XD/6–31 +  + G(2d,2p) level. The dynamic simulations of (pristine and doped) graphene were carried out using CP2K program using cpu10 .

In the following step, the structures were further equilibrated using GROMACS 2020 using the OPLS-AA force field in a simulation box (3 × 3 × 30 nm^3^) in the presence of water molecules (SPC/E water model) in the EM (10 kJ/mole/nm minimum force), NVT (500 ps with 1 fs time steps), NPT (500 ps with 1 fs time steps) and then MD (100 ns with 2 fs time step) simulations. The cutoff radius was adjusted at 1.4 nm for the vdW and Coulomb interactions. We used the Coulomb energy algorithm and Particle Mesh Ewald (PME). The pressure and temperature algorithms were the isotropic Parrinello–Rahman algorithm^63^ at 1 bar and with a nose–hoover (velocity-scaling algorithm in NVT and NPT) at 300 K. The polymer chain lengths were reduced from 30 and 20 nm to 4 and 5 nm for PLGA-PEG and PLGA-PEG-RF, respectively.

### Simulation of self-assembly

All simulations were performed using carbon-based nano-objects with the sheet and spherical geometry in an interface style (representative of the microfluidic environment) and random style (representative of the bulk nanoprecipitation method). All 16 simulations were carried out with the same concentration of drug, polymer, and ACN in simulation boxes with 8 × 8 × 10 nm^3^ sizes using an OPLS-AA force field. The cut-off diameter was set at 1.4 nm for vdW and Coulomb interactions. The Coulomb energy was calculated with a Particle Mesh Ewald (PME) algorithm together with the pressure and temperature algorithms which were an isotropic Parrinello–Rahman algorithm at 1 bar and with the nose–hoover (velocity-scaling algorithm in NVT and NPT) at 300 K. In total, 411 ACN molecules were used for both approaches, alongside 5000 water molecules^64^.

### Gibbs free energy calculation for the self-assembly of NPs

Gibbs free energy was calculated for all samples by the Umbrella sampling technique, as described previously^52^. Briefly, we used the NPs formed from all polymeric NPs produced in the presence of carbon-nanoobjects as Umbrella simulation input structures. This task was completed in two steps: (1) a pull code was applied to separate one chain of the micelle. Afterward, 100 configurations (each as 1 ns) were extracted from the pull code simulation. (2) After applying the pull code for the polymer strand, it was restrained at an increasing center-of-mass (COM) distance from the polymer strands that led to the generation of various configurations for each location. The probability mass function (PMF) curve was extracted in the restrain stage using the polymer strand positions to the COM. In other words, the integration of PMF corresponded to the series of configurations. Finally, Gibbs free energy was obtained by the weighted histogram analysis method (WHAM)^65,66^ for all configurations. During each step, the temperature and pressure algorithms were Berendsen^67^ and the temperature and pressure algorithms were Nose–Hoover and Parrinello-Rahman, respectively. All were employed to the simulation box with a 10 × 10 × 30 nm^3^ size.

### Gibbs free energy calculation for the interaction of the stable sample with a cancer cell membrane

After careful monitoring and screening of the NPs, and to extend the knowledge on the effect of various parameters such as synthesis method (microfluidic or bulk) and percentage of N-doping on the performance of stable NP, Gibbs free energy was calculated for the interaction of the NP with a cancer cell membrane. To this end, a cancer cell membrane molecule was designed through the Charmm-GUI website with DOPC composition. Additionally, the aspect ratio of the selected nano-carbon on the produced particle and consequently its interaction with cancer cell membrane was investigated through Gibbs free energy calculations with similar procedures and algorithms.

## Supplementary Information

Supplementary Information.
