# Supplementary material for: Improving the self-assembly of bioresponsive nanocarriers by engineering doped nanocarbons: a computational atomistic insight
Source: Sci Rep. 2021 Nov 2;11:21538. doi: 10.1038/s41598-021-00817-2 (PMC8564517; doi:10.1038/s41598-021-00817-2)
Supplement: Supplementary file 1 — Supplementary Information. [file 41598_2021_817_MOESM1_ESM.pdf]

Improving the self-assembly of bioresponsive nanocarriers by engineering doped nanocarbons: A computational atomistic insight

Mohammad Khedri [1,2], Nima Beheshtizadeh [3, 4], Reza Maleki [1], Thomas J. Webster [5], and Sima Rezvantalab [6]*

[1] Computational Biology and Chemistry Group (CBCG), Universal Scientific Education and Research Network (USERN), Tehran, Iran.

[2] Department of Chemical Engineering, Amirkabir University of Technology (Tehran Polytechnic), 424 Hafez Avenue, Tehran, Iran.

[3] Department of Tissue Engineering and Applied Cell Sciences, School of Advanced Technologies in Medicine, Tehran University of Medical Sciences, Iran.

[4] Regenerative Medicine Group (REMED), Universal Scientific Education and Research Network (USERN), Tehran, Iran.

[5] Department of Chemical Engineering, Northeastern University, Boston, MA USA

[6] Renewable Energies Department, Faculty of Chemical Engineering, Urmia University of Technology, 57166-419, Urmia, Iran.

[*] Corresponding Author: Dr. Sima Rezvantalab s.rezvantalab@uut.ac.ir

Table S1. Molecular structure and morphology of the carbon nanomaterials used in the simulations.

Geometry	Structure	Total Atom No.	Percent of Total Atoms			
			Carbon	Nitrogen	Boron	Phosphorous
Sheet	Graphene	100	100	-	-	0
	BCN	100	34	33	33	0
	N-doped	100	90	10	-	0
	P-doped	100	50	-	-	50
Spherical	Fullerene	140	100	-	-	-
	BCN	140	33	33	33	-
	N-doped	140	90	10	-	-
	P-doped	140	50	-	-	50

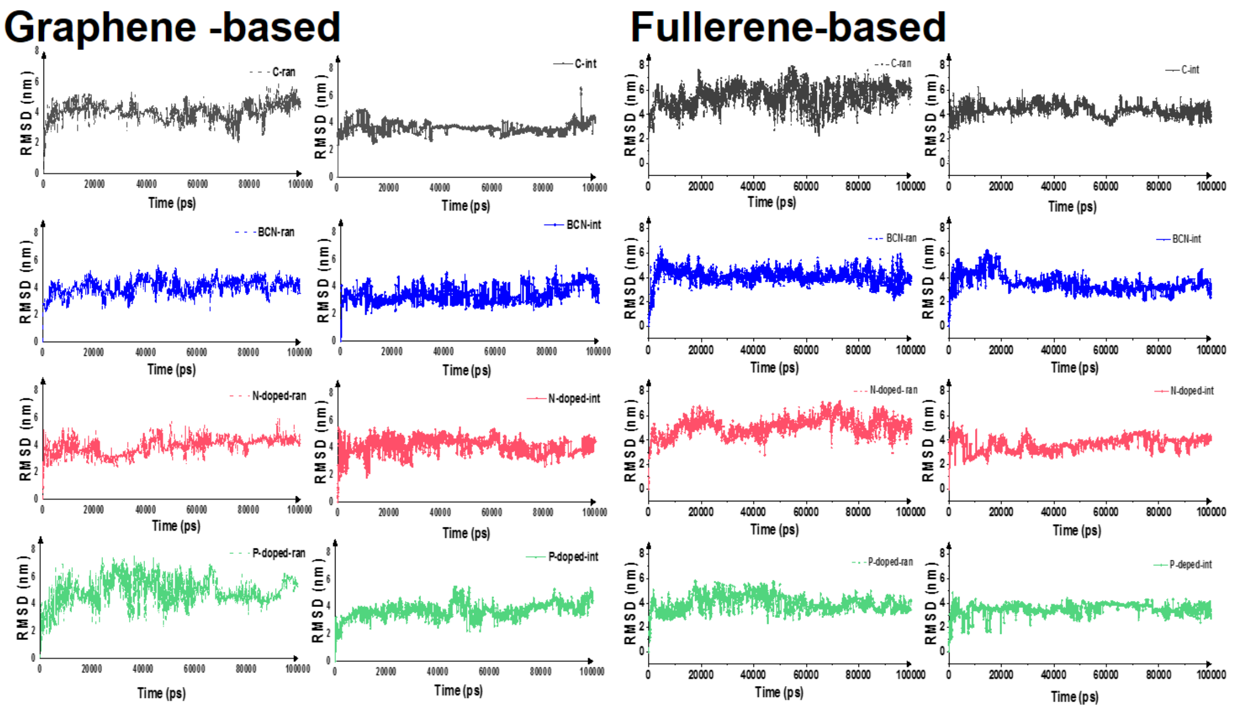

Figure S1. Root mean square deviation (RMSD) fluctuations for the self-assembly of nanoparticles in the presence of nanocarbons

Table S2. Electrostatic and vdW and total energies between the nanocarrier and cancer cell membrane for each case.

Geometry	Structure	Electrostatic (kJ/mole)		vdW (kJ/mole)		Total (kJ/mole)	
		interface	random	interface	random	interface	random
N-doped Sheet	High aspect ratio	-522.616	-414.716	-482.164	-401.621	-1004.8	-816.34
	Low aspect ratio	-713.435	-543.467	-569.216	-519.199	-1282.7	-1062.7
N-doped Spherical	High aspect ratio	-328.547	-315.684	-278.942	-280.247	-607.49	-595.93
	Low aspect ratio	-374.791	-354.161	-291.753	-281.174	-666.54	-635.34
